# Spatiotemporal Dynamics of Dengue in the State of Pará and the Socio-Environmental Determinants in Eastern Brazilian Amazon

**DOI:** 10.3390/idr17040099

**Published:** 2025-08-11

**Authors:** Brenda Caroline Sampaio da Silva, Ricardo José de Paula Souza e Guimarães, Bruno Spacek Godoy, Andressa Tavares Parente, Bergson Cavalcanti de Moraes, Marcia Aparecida da Silva Pimentel, Douglas Batista da Silva Ferreira, Emilene Monteiro Furtado Serra, João de Athaydes Silva Junior, Luciano Jorge Serejo dos Anjos, Everaldo Barreiros de Souza

**Affiliations:** 1 Programa de Pos-Graduação em Ciências Ambientais (PPGCA), Instituto de Geociências, Universidade Federal do Pará, Belém 66075-110, PA, Brazil; brendacaroline444@gmail.com (B.C.S.d.S.); bspacek@ufpa.br (B.S.G.); marciapimentel1989@gmail.com (M.A.d.S.P.); emilene.serra@ig.ufpa.br (E.M.F.S.); ljsanjos@ufpa.br (L.J.S.d.A.); 2 Laboratório de Geoprocessamento, Instituto Evandro Chagas, Ananindeua 67030-000, PA, Brazil; ricardojpsg@gmail.com; 3 Instituto Amazônico de Agriculturas Familiares, Universidade Federal do Pará, Belém 66077-530, PA, Brazil; 4 Instituto de Ciências da Saúde, Universidade Federal do Pará, Belém 67075-110, PA, Brazil; andressatp@ufpa.br; 5 Faculdade de Meteorologia, Programa de Pos-Graduação em Gestão de Risco e Desastre na Amazônia, Instituto de Geociências, Universidade Federal do Pará, Belém 66075-110, PA, Brazil; bergson@ufpa.br (B.C.d.M.); athaydes@ufpa.br (J.d.A.S.J.); 6 Instituto Tecnológico Vale, Belém 67055-090, PA, Brazil; douglas.silva.ferreira@itv.org

**Keywords:** dengue risk modeling, eastern Amazon, spatial epidemiology, deforestation-driven disease risk

## Abstract

Background: The Amazon biome exhibits complex arboviral transmission dynamics influenced by accelerating deforestation, climate change, and socioeconomic inequities. Objectives/Methods: This study integrates official epidemiological records with socioeconomic, environmental, and climate variables by applying advanced geostatistical methods (Moran’s I, SaTScan, kernel density estimation) combined with principal component analysis and negative binomial regression to assess the spatiotemporal dynamics of dengue incidence and its association with socio-environmental determinants across municipalities in Pará state (eastern Brazilian Amazon) from 2010 to 2024. Results: Dengue incidence showed an overall decline but with marked epidemic peaks in 2010–2012, 2016, and 2024. The spatial analysis revealed significant clustering (Moran’s I = 0.221, *p* < 0.01), with persistent high-risk hotspots across most of Pará. Of 144 municipalities, 104 exhibited significant dengue risk, while 58 maintained sustained transmission. Negative binomial regression model identified key determinants: illiteracy, low urbanization, reduced GDP, and climate variables. Conclusions: Dengue transmission in the Amazon is driven by synergistic socio-environmental disruptions, necessitating intersectoral policies that bridge public health surveillance, sustainable land-use governance, and poverty alleviation. Priority actions include targeted vector control in high-risk clusters, coupled with integrated deforestation and climate monitoring to predict outbreak risks. The findings emphasize the urgency of implementing multisectoral interventions tailored to the territorial and socio-environmental complexities of vulnerable Amazonian regions for effective dengue control.

## 1. Introduction

Dengue is the most rapidly spreading vector-borne viral disease globally, with incidence rates increasing approximately 30-fold over the past five decades across more than 100 countries [1]. This arbovirus, transmitted primarily by *Aedes aegypti* and secondarily by *A. albopictus* mosquitoes, both belonging to the family *Culicidae*, comprises four genetically distinct serotypes (DENV-1 to DENV-4), which frequently co-circulate within endemic regions [2]. The global burden of dengue is substantial, with nearly half of the world’s population at risk of infection [3]. Brazil ranks among the most affected countries in the Americas, accounting for over 70% of reported cases in the last decade and posing significant spillover risks to neighboring regions. In 2023 alone, Brazil reported more than 1.6 million suspected dengue cases and 1094 fatalities. By 2024, the country represented over half of all suspected dengue cases in Latin America [4].

The persistence of dengue epidemics in Brazil is influenced by multiple interconnected factors, including high population density, unplanned urbanization, socioeconomic disparities, and the ecological adaptability of mosquito vectors [5]. The ubiquitous presence of *A. aegypti* in urban settings presents a major public health challenge, exacerbating social and economic impacts, particularly among populations with limited access to healthcare, coupled with inadequate housing and sanitation infrastructure [5,6].

Within the Brazilian Legal Amazon, the state of Pará exemplifies a high-risk environment for dengue proliferation due to its unique rural/urban and socioecological conditions. The region’s tropical climate, extensive river networks, rapid demographic expansion, haphazard urban occupation, inequitable distribution of public services, and landscape alterations from land-use changes collectively contribute to increased arbovirus transmission, particularly among vulnerable groups [6,7,8]. Emerging evidence underscores the strong influence of socioecological and climate factors on dengue transmission dynamics in the Amazon [6,9], highlighting the need for integrated analyses to inform targeted intervention strategies [10]. However, regional-scale studies with an interdisciplinary scope that systematically assess these determinants remain scarce, particularly in ecologically, climatologically and socially heterogeneous contexts like the Amazon. Given these gaps, this study aims to analyze the spatiotemporal dynamics of dengue incidence in Pará state from 2010 to 2024, evaluating its association with socio-environmental determinants at the municipal level. By identifying localized drivers of transmission and high-risk areas, our findings can support scientific evidence-based surveillance strategies and public health policies tailored to the Amazonian context, ultimately contributing to more effective dengue control in the region.

## 2. Materials and Methods

**Figure 1:**
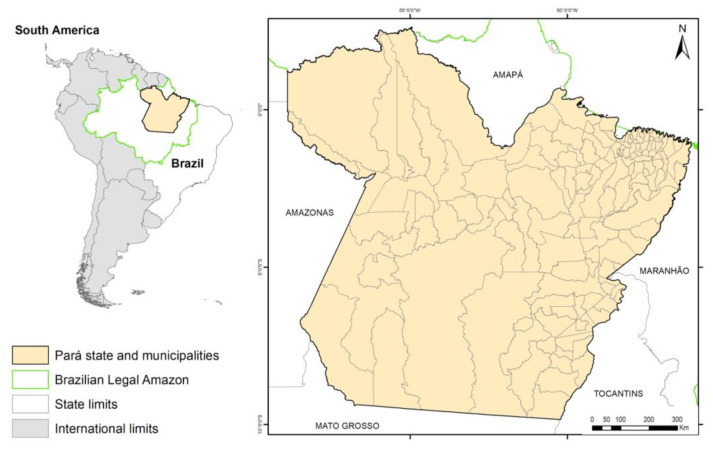
Study area: the state of Pará and its municipalities.

This study was conducted in the state of Pará, located in the eastern Brazilian Legal Amazon (Figure 1). Covering approximately 1,246,000 km^2^ and comprising 144 municipalities, Pará exhibits significant socio-environmental diversity [11]. As the most populous state in northern Brazil, its population increased from 6.8 million in 2004 to 8.7 million in 2024 [12]. The regional climate is characterized by two distinct seasons: a rainy season (January to May) with heavy precipitation (300 to 400 mm/month) and an average temperature around 26 °C, and a dry season (June to December) with reduced rainfall (<100 mm/month) and higher temperatures (27 to 28 °C) [13,14]. Dengue incidence predominantly occurs during the rainy regime, accounting for 69% of annual cases, when regional climate conditions maximize vector proliferation [9].

We compiled multidisciplinary data across health, socioeconomic, environmental, and climate dimensions for all 144 municipalities throughout the state of Pará. The complete list of variables and their respective dimensions and units is detailed in Table 1. Epidemiological data included positive dengue case records from the Brazilian Notifiable Diseases Information System (SINAN), Ministry of Health/DATASUS [15] and mortality data from the Mortality Information System (SIM). Health infrastructure was assessed using the National Registry of Health Establishments [16]. From the official platform of the National Demographic Census made available by the Brazilian Institute of Geography and Statistics [17], we acquired most of the social and economic data: total population and by age range, population density, household sanitation (water supply, sewage, waste disposal), GDP per capita, and illiteracy rates. The Municipal Human Development Index (MHDI) was extracted from [18]. Environmental data included urbanized municipal areas [19] and annual deforestation rates from the PRODES/INPE [20]. Climate variables comprised maximum/minimum air temperatures from Climate Prediction Center/National Centers for Environmental Prediction (CPC/NCEP), an interpolated gridded data using global weather stations [21]. We also used precipitation data from Climate Hazards Group InfraRed Precipitation with Station (CHIRPS) from University of California Santa Barbara (UCSB), combining satellite and ground-station data [22]. All datasets were processed and harmonized to municipal-level spatial resolution and annual/monthly temporal scales from 2010 to 2024.

The spatial patterns of dengue incidence across municipalities in Pará were analyzed by applying the Moran Index (Moran’s I) and the Local Moran Index (LISA). Moran’s I was applied to assess spatial autocorrelation at both global and local scales [23], under the assumption of uniform dengue infection risk and a Poisson-distributed dissemination process. The null hypothesis posits a random spatial distribution of cases. Spatial weights were computed based on queen-type contiguity adjacency. Moran’s I values range from −1 (perfect dispersion) to 1 (perfect correlation), with 0 indicating spatial randomness. LISA complemented this analysis by identifying localized clusters and outliers, decomposing global spatial dependence into region-specific patterns [24]. Statistically significant clusters (α = 0.05) were classified into four categories, as follows. High–High (HH): High-incidence municipalities surrounded by high-incidence neighbors; Low–Low (LL): Low-incidence municipalities adjacent to other low-incidence areas; High–Low (HL) and Low–High (LH): Spatial outliers where high- and low-incidence units were surrounded by dissimilar values, respectively.

We also employed the Kulldorff spatial scan statistic (SaTScan) to detect spatiotemporal clusters using a dynamic circular window [25]. The null hypothesis assumed random dengue distribution, while the alternative suggested elevated incidence within the scanning window. The log-likelihood ratio (LLR) was computed via Monte Carlo simulations, with statistical significance assessed at *p* < 0.05. A Poisson model was selected, given its suitability for count data with known population-at-risk denominators [6,23]. Relative risk (RR) was calculated to compare intra- versus extra-cluster incidence, as follows. RR = 1: No risk difference; RR > 1: Elevated risk within the cluster; and RR < 1: Reduced risk (potential protective effect). Persistent clusters across time intervals were identified using map algebra geostatistics and kernel density estimation, whose method allowed the integration of overlapping high-risk zones [26].

Furthermore, the relationship between the number of dengue cases and socio-environmental factors was analyzed using Generalized Linear Models (GLMs) with Poisson and Negative Binomial distributions (log link function), with the latter applied in cases of overdispersion [27]. Predictor variables were organized into four thematic blocks (socioeconomic, health, environmental/climatic, and sanitary infrastructure) and standardized as z-scores. Principal Component Analysis (PCA) was applied individually to each block for dimensionality reduction, retaining components that explained more than 70% of the cumulative variance [28]. Model selection was based on the Akaike Information Criterion (AIC). Multicollinearity was assessed through the Variance Inflation Factor (VIF), and model fit quality was evaluated using Zhang’s R^2^ and graphical analysis of residuals (Randomized Quantile Residuals—RQR) [29]. To assess the spatial dependence of the residuals, Moran’s Global I statistic was calculated based on Pearson residuals, using a queen contiguity spatial weight matrix. The absence of significant spatial autocorrelation in the residuals indicated the spatial adequacy of the adjusted model. Finally, it is important to emphasize that the analytical approach employed in this study was inferential and explanatory, aiming to understand the patterns of association between socio-environmental variables and dengue occurrence, rather than to develop a predictive model. All data analysis and results generation were performed in RStudio version 2025.05.0 (Posit, Reykjavík, Iceland).

## 3. Results

**Figure 2:**
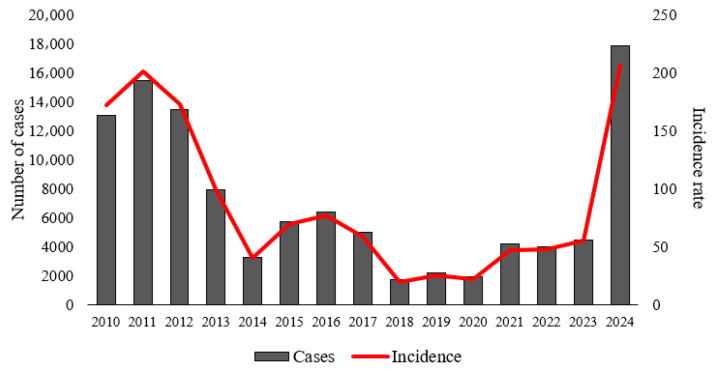
Time series of the annual total of confirmed cases and incidence rate of dengue in municipalities of the state of Pará.

Figure 2 illustrates that the state of Pará recorded 106,705 confirmed dengue cases over the 15-year study period, with the most critical years being 2010 (13,055 cases; 172 cases per 100,000 inhabitants), 2011 (15,484 cases; 201 cases per 100,000 inhabitants), 2012 (13,482 cases; 173 cases per 100,000 inhabitants), and 2024 (17,881 cases; 206 cases per 100,000 inhabitants).

**Figure 3:**
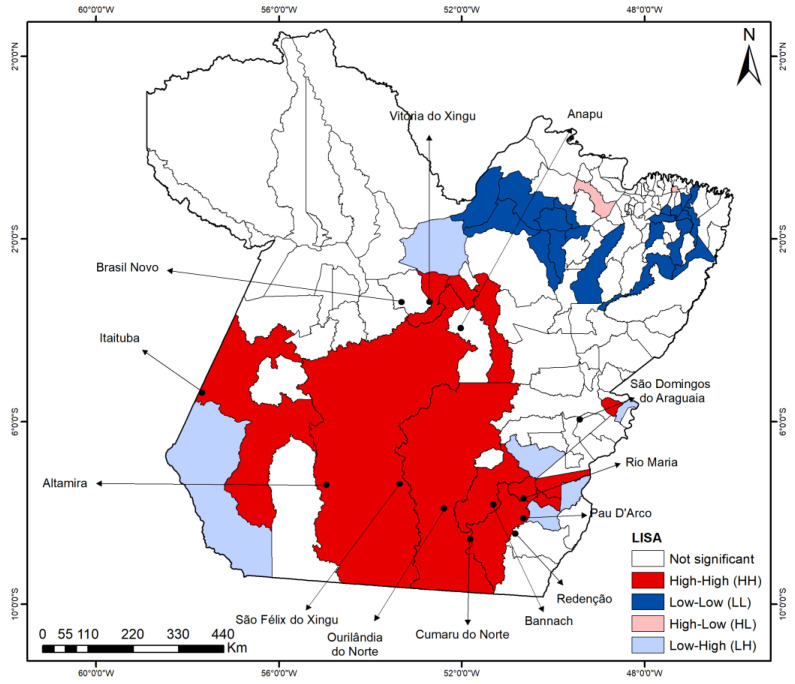
LISA clusters for dengue incidence rates in the municipalities of the state of Pará (2010–2024).

Spatial autocorrelation in dengue incidence rates was confirmed by global Moran’s I (I = 0.221, *p* < 0.001). The LISA map (Figure 3) revealed a prominent high–high (H-H) cluster spanning southern Pará, encompassing 13 municipalities: Itaituba, Altamira, São Félix do Xingu, Ourilândia do Norte, Redenção, Pau D’arco, Bannach, Rio Maria, Anapu, Vitória do Xingu, Brasil Novo, Cumaru do Norte and São Domingos do Araguaia. Additionally, two low–low (L-L) clusters were identified in the northeastern region, comprising 20 municipalities.

The quantitative results obtained by SaTScan are shown in Table 2. This analysis objectively detected 41 statistically significant spatiotemporal clusters (*p* < 0.05), thus indicating dengue transmission risk throughout the state of Pará. The highest relative risk (RR) was observed in Cluster 1 (RR = 100.15), followed by Cluster 15 (RR = 38.17) and Cluster 5 (RR = 15.07), primarily covering Belterra (Cluster 1 and 5) and Sapucaia. Cluster 2 exhibited the broadest spatial extent, involving 73 municipalities, suggesting extensive transmission connectivity across the state of Pará. All years contained significant clusters, reinforcing persistent hotspots.

**Figure 4:**
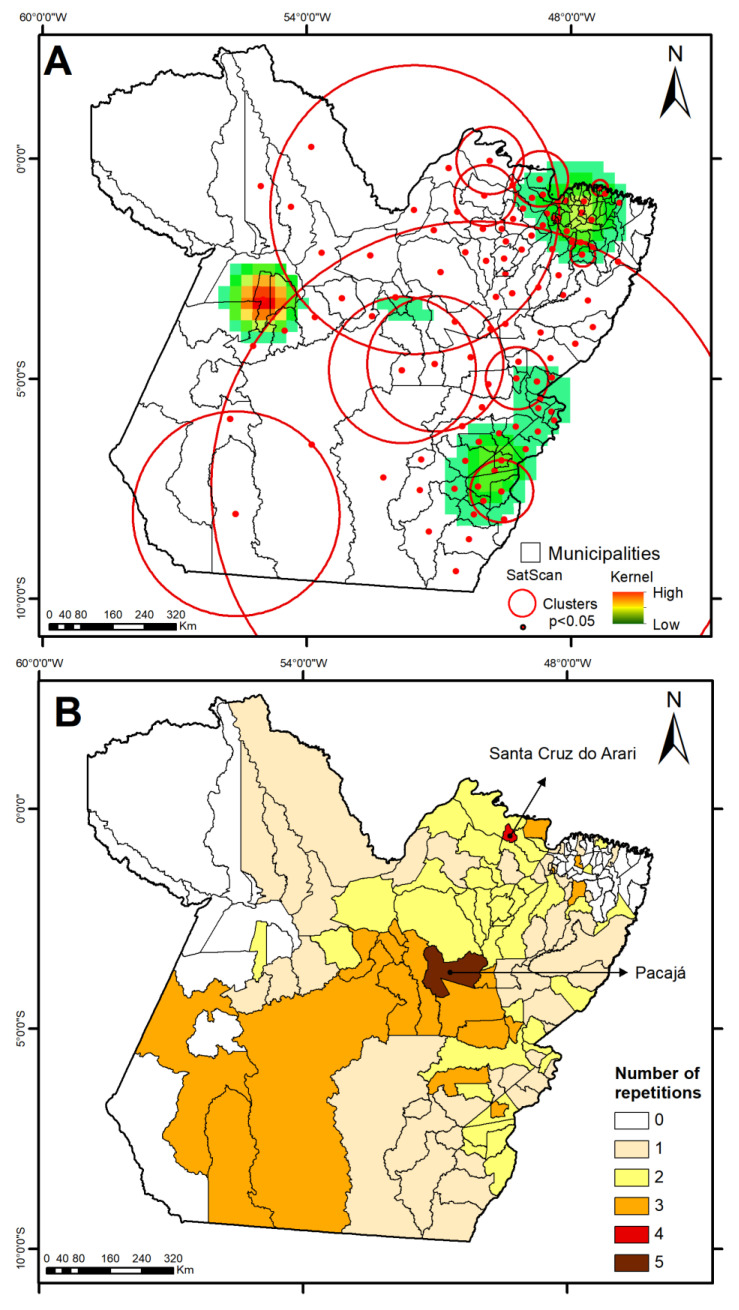
Spatial patterns through Kernel density estimation in municipalities of the state of Pará (2010–2024) from (**A**) SaTScan and (**B**) persistent risk of dengue (number of repetitions).

With the use of map algebra geostatistics and kernel density estimation, Figure 4 was produced, enabling a comprehensive spatial analysis of dengue risk in terms of cluster overlap and disease persistence in municipalities along the region, taking into account the years 2010–2024. These maps highlight the presence of elevated dengue risk concentrations in northeastern, southeastern, and part of the central-western regions of the state of Pará, as indicated in regions containing medium to high kernel values (Figure 4, top). Of the state’s 144 municipalities, 104 (72.2%) exhibited spatiotemporal dengue risk clusters, with 58 municipalities showing persistent transmission patterns. Notably, two municipalities (Santa Cruz do Arari and Pacajá) presented significant clusters in 4 and 5 distinct periods, respectively, indicating hotspots with sustained epidemiological vulnerability (Figure 4, bottom).

In Table 3, the integrated analysis of dengue epidemiology (cases and incidence rate) with socioeconomic, environmental, and climate variables revealed substantial heterogeneity along the state of Pará, with 68% of households lacking adequate sewage systems, 8% facing deficient water supply, and 34% not having proper waste collection services. Additionally, the illiteracy rate reaches 30%, and per capita Gross Domestic Product (GDP) exhibits extreme values, highlighting pronounced socioeconomic disparities. Climate conditions further exacerbated transmission risk, with mean precipitation (207 mm) and air temperature (23 to 32 °C) fostering vector proliferation.

**Figure 5:**
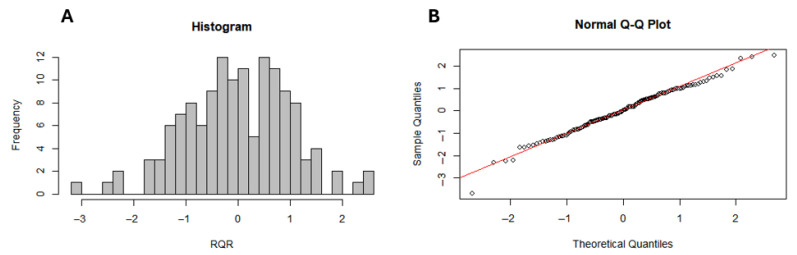
Model residual diagnostics: Histogram of the distribution of Randomized Quantile Residuals (RQR), used to assess the adequacy of the residuals’ distribution relative to the model assumptions (**A**) and QQ plot of the RQR, comparing observed versus theoretical quantiles to evaluate potential deviations from residual normality (**B**).

The negative binomial regression model (AIC = 2001) outperformed the Poisson model (AIC = 94,972), confirming its superior fit for assessing socio-environmental drivers. Multicollinearity analysis indicated that the Variance Inflation Factor (VIF) was below 5 for all principal components, evidencing the absence of significant multicollinearity. Additionally, the model presented a Zhang’s R^2^ of 0.19, indicating a moderate fit. The analysis of randomized quantile residuals (RQR) showed that the histogram does not exhibit a perfectly normal pattern, reflecting some asymmetry or dispersion in the residuals. In the QQ plot, a slight deviation of the points from the reference line was observed, especially at the extremes, indicating the presence of outlier residuals. Despite this, there is no clear evidence of severe departures from the expected residual distribution, suggesting that the model presents an acceptable fit (Figure 5). Furthermore, spatial autocorrelation of model residuals was assessed using Global Moran’s I (I = −0.0339; *p* = 0.7147), indicating no significant spatial dependence and suggesting that the model adequately accounted for the spatial structure inherent in the data.

Table 4 shows that the blocks that presented a significant effect (*p* < 0.05) on the number of dengue cases include socioeconomic_PC1 and environmental_PC. By evaluating the coefficients representing the contribution of each original variable to the principal components generated by the analysis, it was observed that the economic block was synthesized into two principal components (PC1: 60.14% and PC2: 24.30%, totaling 84.45% of cumulative variance explained). The first component was associated with illiteracy (loading: 0.61), urban area (−0.60), and GDP (−0.50), while the second component was strongly correlated with GDP (−0.86). The health block was represented by two principal components (PC1: 56.22% and PC2: 33.33%, explaining 89.55% of the variance). PC1 was mainly associated with mortality rate (−0.70) and the total number of health facilities (−0.71), whereas PC2 was predominantly correlated with the proportion of the population aged over 60 years (−0.99). The environmental block was summarized into two principal components (PC1: 64.83% and PC2: 18.23%, explaining a cumulative 83.06%). PC1 was related to accumulated precipitation (0.55) and maximum temperature (−0.52), while PC2 showed correlations with minimum temperature (−0.70) and deforestation (−0.58). Lastly, the sanitation block was represented by two principal components (PC1: 61.47% and PC2: 26.33%, totaling 87.79% of the variance explained). PC1 was associated with uncollected waste (−0.65) and inadequate water supply (−0.58), while PC2 was correlated with inadequate sewage services (0.83) and with inadequate water supply (−0.55).

## 4. Discussion

This study examines the evolution of dengue transmission in the state of Pará, in the Brazilian Amazon, over a 15-year period, integrating spatiotemporal analyses at the municipal level to assess associations with socio-environmental determinants. To our knowledge, this is the first study in Pará to adopt an interdisciplinary approach, combining geospatial epidemiology with socio-environmental factors that drive the spread of arboviruses.

A declining trend in dengue incidence was observed across Pará, with incidence peaks occurring between 2010 and 2012 and again in 2024. Similar patterns have been reported in other states of the Brazilian Legal Amazon [6] and across Latin America [4,5,6,7,8,9,10,11,12,13,14,15,16,17,18,19,20,21,22,23,24,25,26,27,28,29,30], where large-scale epidemics coincided with these periods. Several hypotheses have been discussed to explain such outbreaks, including insecticide resistance and increases in average temperatures caused by climate change [4]. Pará remains highly vulnerable to dengue due to its tropical climate, intense rainfall, inadequate sanitation, high population density, and accelerated deforestation—factors that reflect significant land-use changes [11,20,31,32,33]. Furthermore, the Amazon region has experienced an increase in dengue lethality, rising from 0.02% (2001–2005) to 0.07% (2016–2021) [6]. Underreporting remains a critical issue, with estimates suggesting that only 1 in every 20 confirmed cases is officially recorded in the National System for Notifiable Diseases (SINAN) [4,5,6,7,8,9,10,11,12,13,14,15,16,17,18,19,20,21,22,23,24,25,26,27,28,29,30,31,32,33,34].

Spatial analysis identified significant high-incidence clusters (LISA, Kernel, and SaTScan), concentrated in the northeastern, southeastern, and most of the central-western regions of the state of Pará. These findings indicate that dengue dissemination does not occur uniformly, but follows territorial patterns linked to specific ecological and socio-structural contexts. Municipalities such as Altamira, São Félix do Xingu, Itaituba, and Anapu, leaders in deforestation rates within the Brazilian Legal Amazon [20], were consistently classified as high–high clusters. Even regions outside the agricultural frontier, such as northeastern Pará, exhibited high vulnerability, resulting from the combination of forest cover loss, agricultural expansion, and deficient urban infrastructure [35,36].

These results reinforce that dengue control in the Amazon requires integrated policies that coordinate environmental management with urban infrastructure development and public health actions. Environmental degradation, particularly deforestation and the unregulated expansion of agricultural frontiers, amplifies epidemiological risks by altering ecosystems and creating new environments conducive to vector proliferation [35]. Containment policies must adopt an intersectoral approach, integrating deforestation control, the expansion of basic sanitation services, and sustainable urban planning.

The large-scale clusters detected by SaTScan, such as Cluster 2 (73 municipalities) and Cluster 3 (40 municipalities), suggest that population mobility and transportation networks play a central role in transmission connectivity [37]. Even municipalities with distinct structural profiles can be rapidly affected by outbreaks originating in neighboring areas. Thus, surveillance and response strategies must be planned within interconnected territorial blocks, overcoming isolated, municipality-based approaches.

The persistence of clusters across multiple periods also reveals the presence of “transmission nodes”, i.e., municipalities with perennial vector populations sustained by chronic structural vulnerabilities. Municipalities such as Pacajá and Santa Cruz do Arari, identified with clusters in four or five distinct periods, should be treated as priority areas in containment strategies, with permanent vector monitoring, community education campaigns, and prioritized investments in basic infrastructure.

The negative binomial regression model indicated that two principal components had significant effects: socioeconomic_PC1 and environmental_PC1. The socioeconomic_PC1 component synthesized a context of high social vulnerability, heavily influenced by illiteracy (0.61), low urbanization (−0.60), and reduced per capita GDP (−0.50). This component reflects a scenario where limited individual capacity for adopting preventive measures, combined with precarious urban infrastructure, increases the risk of vector proliferation and sustained transmission [38]. This combination indicates that, in municipalities with these characteristics, punctual and emergency interventions will have limited impact unless chronic structural vulnerabilities are addressed.

Conversely, environmental_PC1 grouped municipalities with high rainfall volumes (0.55) and moderately mild maximum temperatures (−0.52), climatic conditions known to favor the maintenance of breeding sites and extend vector longevity. The combination of these variables suggests that regions with intense hydrological cycles and temperatures conducive to *Aedes aegypti* activity face elevated epidemiological risks [30].

The inferential model achieved an explanatory power of 19% (Zhang’s pseudo-R^2^), a moderate value, yet expected in complex, multicausal, and spatially dependent phenomena such as dengue. Incorporating additional variables, such as population mobility, vector presence data, climatic lags, and detailed land-use metrics, could enhance explanatory capacity. Moreover, employing models capable of capturing spatial variation in explanatory effects, such as Geographically Weighted Regression (GWR), represents a promising methodological alternative to account for spatial heterogeneity.

Despite its contributions, several limitations must be acknowledged. This study relied on secondary data, which are subject to underreporting and registration inconsistencies, especially in areas with limited epidemiological surveillance capacity. Furthermore, conducting analyses at the municipal scale may obscure internal heterogeneities, limiting the detection of critical micro-areas. Future research should advance to finer spatial scales, such as census tracts or neighborhood units, and integrate connectivity and mobility variables, thereby enhancing sensitivity in identifying local patterns and dynamics.

## 5. Conclusions

This study provides a comprehensive analysis of dengue fever dynamics in the Brazilian Amazon, integrating spatiotemporal epidemiology with socio-environmental determinants to identify persistent transmission patterns across municipalities within the state of Pará from 2010 to 2024. Using advanced geostatistical methods (Moran’s I, SaTScan, kernel density estimation) combined with principal component analysis and negative binomial regression, we demonstrate how deforestation, regional climate variability, and structural vulnerabilities synergistically drive dengue hotspots in this ecologically complex region. Our findings reveal significant spatial autocorrelation, with high-risk clusters concentrated in 72.2% of municipalities, particularly in deforestation frontiers like Altamira and São Félix do Xingu. The identification of perennial “transmission nodes” (e.g., Pacajá and Santa Cruz do Arari) underscores the long-term ecological persistence of arboviral transmission in areas undergoing rapid land-use change.

The methodological approach, including PCA-driven regression validated by robust spatial statistics (e.g., residual autocorrelation testing), provide a replicable framework for arboviral risk mapping in heterogeneous tropical landscapes within Amazon basin. However, the moderate explanatory power of our model (Zhang’s R^2^ = 0.19) signals the complexity of dengue’s socio-ecological drivers, urging future research to incorporate finer-scale data, such as mobility networks, land-use transitions, and time-lagged deforestation effects, in order to better capture delayed feedback loops in Amazonian ecosystems. Integrating these dynamics with predictive modeling (e.g., GWR or machine learning) could refine early-warning systems and optimize resource allocation across the region’s rapidly changing frontier.

Ultimately, our work bridges critical gaps between environmental governance and public health in the Amazon, advocating for policies that couple deforestation control with urban infrastructure investment. The persistent clusters identified here serve as sentinel sites for monitoring climate–arbovirus interactions, while the interdisciplinary approach sets a precedent for studying emerging tropical diseases in tropical deforestation hotspots. As climate change amplifies vector suitability, these insights demand urgent translation into adaptive policies that prioritize both ecological integrity and health equity in the Amazon’s vulnerable communities.

## Figures and Tables

**Table 1:** Complete list of variables and their respective dimensions (blocks), descriptions, and units.

Dimension	Variable	Description	Unit
Epidemiological	Dengue cases	Positive cases of dengue	Total count
	Dengue incidence	(Number of dengue cases ÷ Population) × 100,000 inhabitants	Dimensionless
Health	Health establishments	Number of hospitals or health centers (total in December of each year)	Total count
	Mortality < 14 years	Total number of deaths of people under 14 yr age	Total count
	Population_60+	Population over 60 years old	%
Socioeconomic	Urban area	Size of urbanized area	km^2^
	Illiterate	People aged 15 or over who are illiterate	%
	GDP	Gross domestic product per capita	R$
Sanitary	Inadequate sewage system	Households with sewage system via a rudimentary septic tank or hole, via a ditch, via a river, lake, or stream and without bathroom	%
	Inadequate waste collection	Households that do not have waste collection	%
	Inadequate water supply	Households whose water supply comes from stored rainwater, rivers, dams, streams, or creeks	%
Environmental/ Climate	Deforestation	Deforestation increase per year	km^2^
	Min. and Max. air temperature	Minimum and Maximum air temperature	°C
	Precipitation	CHIRPS accumulated precipitation	mm

Note: The dengue cases variable was used as the response variable in the GLM. Dengue incidence was calculated for geostatistical analyses. The variables from the Health, Socioeconomic, Sanitary, and Environmental/Climate blocks were used for PCA, and their principal components were used as response variables in the GLM.

**Table 2:** Characteristics of statistically significant clusters (*p* < 0.05) detected by the SaTScan regarding dengue risk in municipalities of the state of Pará (2010–2024).

Cluster	Period	Population	Municipalities	Observed Cases	Expected Cases	Relative Risk
1	2010 to 2016	17,385	1	9239	100.90	100.15
2	2010 to 2013	3,514,901	73	29,624	11,176.26	3.28
3	2024	3,785,011	40	9211	3354.04	2.91
4	2017 to 2022	140,630	6	3183	738.49	4.41
5	2018 to 2022	17,385	1	1144	76.69	15.07
6	2010 to 2012	62,468	2	1328	151.41	8.87
7	2016 to 2017	783,960	8	3922	1354.87	2.97
8	2022	207,426	1	1194	227.48	5.30
9	2020 to 2022	24,254	1	600	64.27	9.38
10	2010 to 2012	122,108	1	1140	286.54	4.01
11	2010 to 2012	80,020	4	916	188.60	4.89
12	2016	57,208	1	482	49.35	9.81
13	2020 to 2021	246,419	3	1372	426.43	3.25
14	2016 to 2017	15,407	1	316	26.61	11.91
15	2015	5635	1	183	4.80	38.17
16	2019	42,763	1	266	31.88	8.36
17	2023	150,196	5	433	114.78	3.78
18	2016 to 2022	21,859	1	474	135.74	3.50
19	2022	24,438	1	178	20.96	8.51
20	2018	27,746	1	172	24.20	7.12
21	2015	27,345	1	166	23.80	6.98
22	2016	24,254	1	139	20.74	6.71
23	2018	30,379	1	149	25.67	5.81
24	2021	29,475	1	127	26.71	4.76
25	2015	31,320	2	125	27.50	4.55
26	2018 to 2020	31,050	1	231	82.52	2.80
27	2015	28,418	1	115	25.07	4.59
28	2023	246,419	3	456	248.49	1.84
29	2019	186,989	6	338	166.26	2.04
30	2022	37,795	1	109	32.86	3.32
31	2015	122,108	1	224	105.67	2.12
32	2016	60,091	2	133	52.57	2.53
33	2017	51,377	1	116	44.94	2.58
34	2019	15,497	1	59	14.34	4.12
35	2017	20,837	1	66	17.92	3.68
36	2021	44,444	1	107	41.54	2.58
37	2014	15,407	1	54	13.19	4.10
38	2021	15,407	1	43	13.61	3.16
39	2020	7209	1	26	6.35	4.09
40	2021	59,360	1	96	52.44	1.83
41	2022	37,222	2	68	34.52	1.97

**Table 3:** Descriptive statistics of dengue data and socioeconomic, environmental, and climate variables.

Variables	Mean	Median	Std. Deviation	Minimum	Maximum
Number of dengue cases	585.61	187	1227.80	0	10,387
Dengue incidence rate	135.34	41.47	412.68	0	4643.11
Number of health establishments	142.05	78.82	303.17	17.73	3431.73
Mortality under 14 years	281.65	162.50	537.04	10	5568
Population_60+	10.46	10.18	2.42	5.09	16.29
Urban area (km^2^)	12.12	7.35	17.27	0.83	147.35
Illiterate population up to 15 years old	30.72	29.07	11.78	6.94	64.89
GDP	28,067.05	13,844.21	80,160.77	6447.28	894,806.28
Households with inadequate sewage	68.55	74.08	19.30	9.12	98.04
Households that do not have waste collection	34.40	33.34	19.060	1.8316	78
Households with inadequate water supply	8.94	2.99	14.84	0.08	77.05
Accumulated deforestation (km^2^)	21.56	2.39	53.88	0	415.29
Average minimum temperature (°C)	23.07	23.09	0.81	20.97	24.89
Average maximum temperature (°C)	31.87	31.87	0.78	30.38	33.66
Accumulated precipitation (mm)	207.66	208.89	38.27	139.07	278.40

**Table 4:** Blocks selected through PCA and analyzed using Negative Binomial distribution.

Coefficient	Estimated	Standard Error	z-Value	*p*-Value
Intercept	6.23954	0.09311	67.010	0.0000 ^1^
socioeconomic_PC1	−0.47641	0.12038	−3.958	0.0006 ^1^
socioeconomic_PC2	0.13009	0.14263	0.912	0.3617
health_PC1	−0.12538	0.10262	−1.222	0.2218
health _PC2	−0.08114	0.12727	−0.63	0.5238
environmental_PC1	−0.15828	0.07781	−2.034	0.0419 ^1^
environmental_PC2	−0.21441	0.13204	−1.624	0.1044
sanitary_PC1	−0.02620	0.11667	−0.225	0.8223
sanitary_PC2	0.08907	0.12684	0.702	0.4825

^1^ *p*-value > 0.05.

## Data Availability

The databases and their respective sources and references were described in the Materials and Methods section.
